# Osteonecrosis of the Femoral Head: Etiology, Investigations, and Management

**DOI:** 10.7759/cureus.3171

**Published:** 2018-08-21

**Authors:** Shakoor A Baig, MN Baig

**Affiliations:** 1 Trauma & Orthopaedics, Leicester Royal Infirmary, Leicester, GBR; 2 Trauma & Orthopaedic Surgery, Galway University Hospital, Galway, IRL

**Keywords:** osteonecrosis, bisphosphonates, hyperbaric oxygen, core decompression

## Abstract

Femoral head osteonecrosis is a condition caused by a compromise of the blood supply of the femoral head. The precarious blood supply of the head and its role as a major weight-bearing joint makes it one of the most common bones to be affected by osteonecrosis. We describe the etiology, clinical presentation, investigations and common management options used nowadays to treat it.

## Introduction and background

Osteonecrosis (ON) or avascular necrosis of the femoral head is a condition characterized by the death of osteocytes and bone marrow. Osteonecrosis is caused by an inadequate blood supply to the affected segment of the subchondral bone and is sometimes called “coronary disease of the hip” because it simulates the ischemic condition of the heart.

## Review

Osteonecrosis of the femoral head (ONFH) usually affects young adults in the third and fourth decades of life. There has been an increasing trend in its diagnosis; every year about 10,000 to 20,000 new cases are diagnosed in the United States [1]. According to the literature, about 5%-12% of hip arthroplasties are performed for the treatment of this condition per annum [2]. It starts with one femoral head being affected first; bilateral involvement occurs in two years in 72% of cases [2].

Etiology and pathophysiology

There are two groups of patients diagnosed with hip osteonecrosis: (a) patients with no apparent etiological or risk factor and (b) patients with clearly identified etiology. Based on the etiology, osteonecrosis can be idiopathic (primary) or secondary.

There are multiple contributory etiological factors of osteonecrosis. The use of glucocorticoids and excessive alcohol intake is associated with more than 80% of atraumatic cases. ONFH is the result of the combined effects of genetic predisposition, metabolic factors, and local factors affecting blood supply (e.g., vascular damage, increased intraosseous pressure, and mechanical stresses) [3-4].

**Figure 1:**
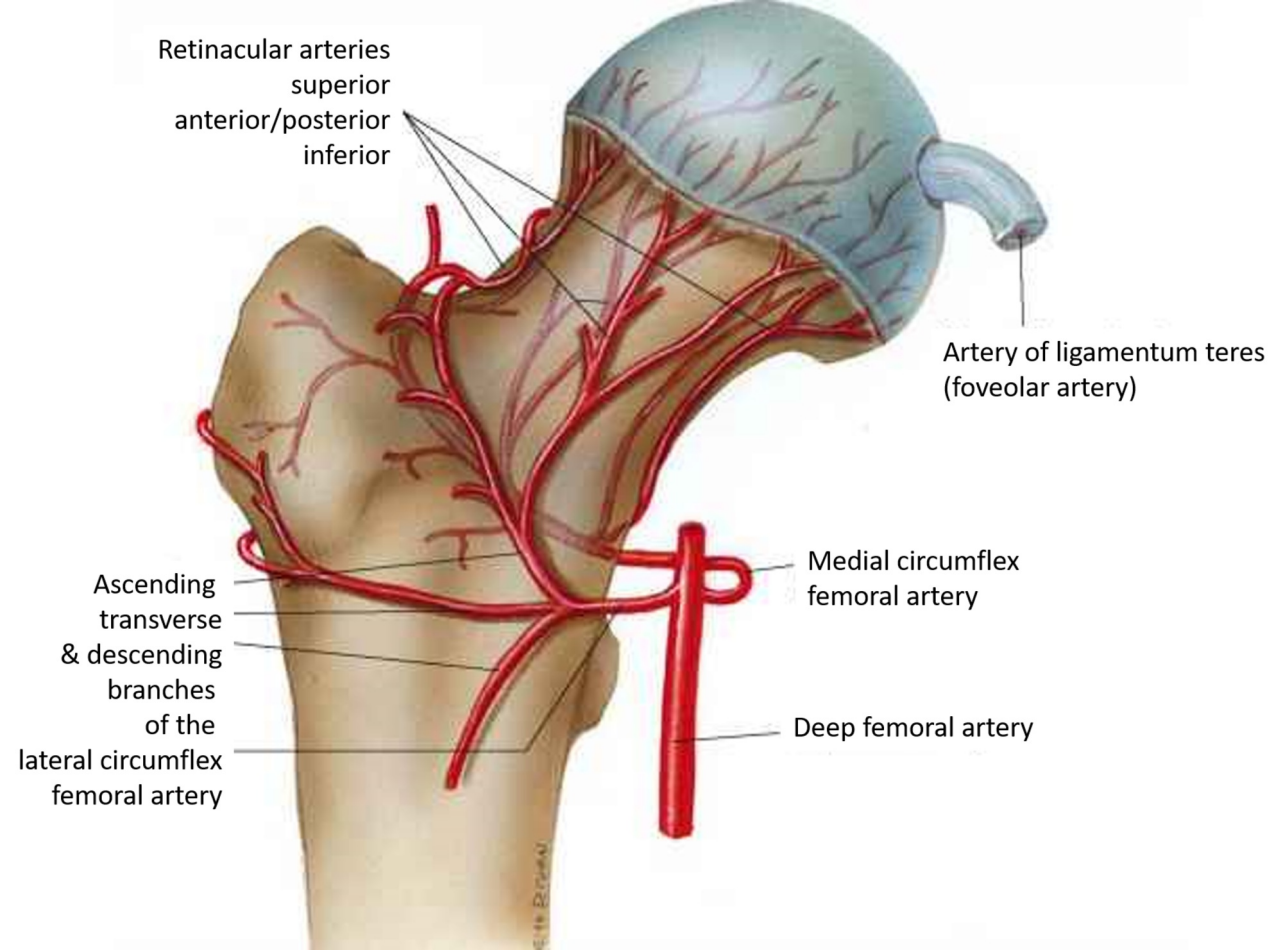
Femoral head blood supply Femoral head and neck blood supply (Courtesy ALPF Medical Research)

The vascular anatomy varies, but a majority of the population has a lateral femoral circumflex artery, which gives rise to three or four branches (i.e., the retinacular vessels); an obturator artery that gives rise to the vessels within the ligamentum teres; and an ascending branch of the medial femoral circumflex artery, which supplies the greater trochanter and anastomoses with the lateral femoral circumflex artery (Figure 1) [5].

In cases of trauma, the resulting osteonecrosis is better understood because the vascularity around the femoral head is severely disturbed as a result of the injury. The most common traumatic causes of OFNH are dislocations and fractures. About 15%-50% of displaced femoral neck fractures and 10%-25% of hip dislocations end in ONFH [6].

In the cases of ONFH cited in the literature, elevated intraosseous pressure has been measured secondary to venous outflow obstruction and venous stasis. In the studies measuring intraosseous pressure, the bone is considered a closed compartment and pressure increases due to the compromised blood supply in many conditions that are known to causes OFNH.

Clinical presentation 

ONFH may be asymptomatic in the early stages. Clinically, the most common symptom is a deep pain in the groin. Pain may be referred to the ipsilateral buttock or knee. Symptoms worsen with weight bearing and are relieved with rest. The range of motion becomes limited, particularly hip abduction and internal rotation; logrolling (i.e., passive internal and external rotation) elicits pain [6].

Imaging

The imaging modalities that help in the diagnosis of OFNH are plain anteroposterior and lateral “frog-leg” radiographs, computed tomography (CT) scans, and magnetic resonance imaging (MRI). Plain radiographs are the first imaging investigation given their low cost, simplicity, and availability. The disadvantage of radiographs is their insensitivity for detecting ON in its early stages.

**Figure 2:**
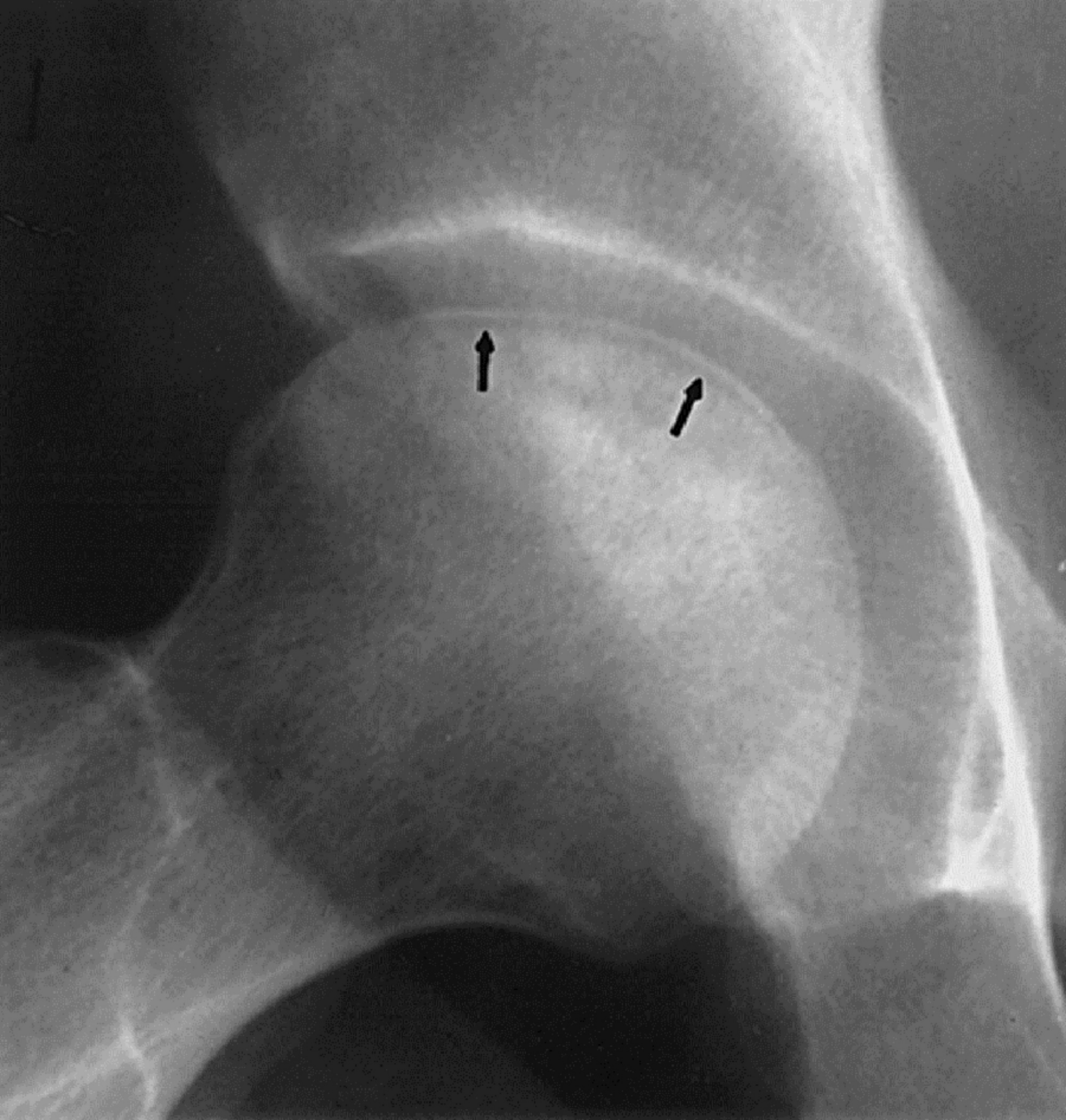
X-ray hip Crescent sign. Arrows showing the hypointense crescent. (Courtesy http://onradiology.blogspot.com)

**Figure 3:**
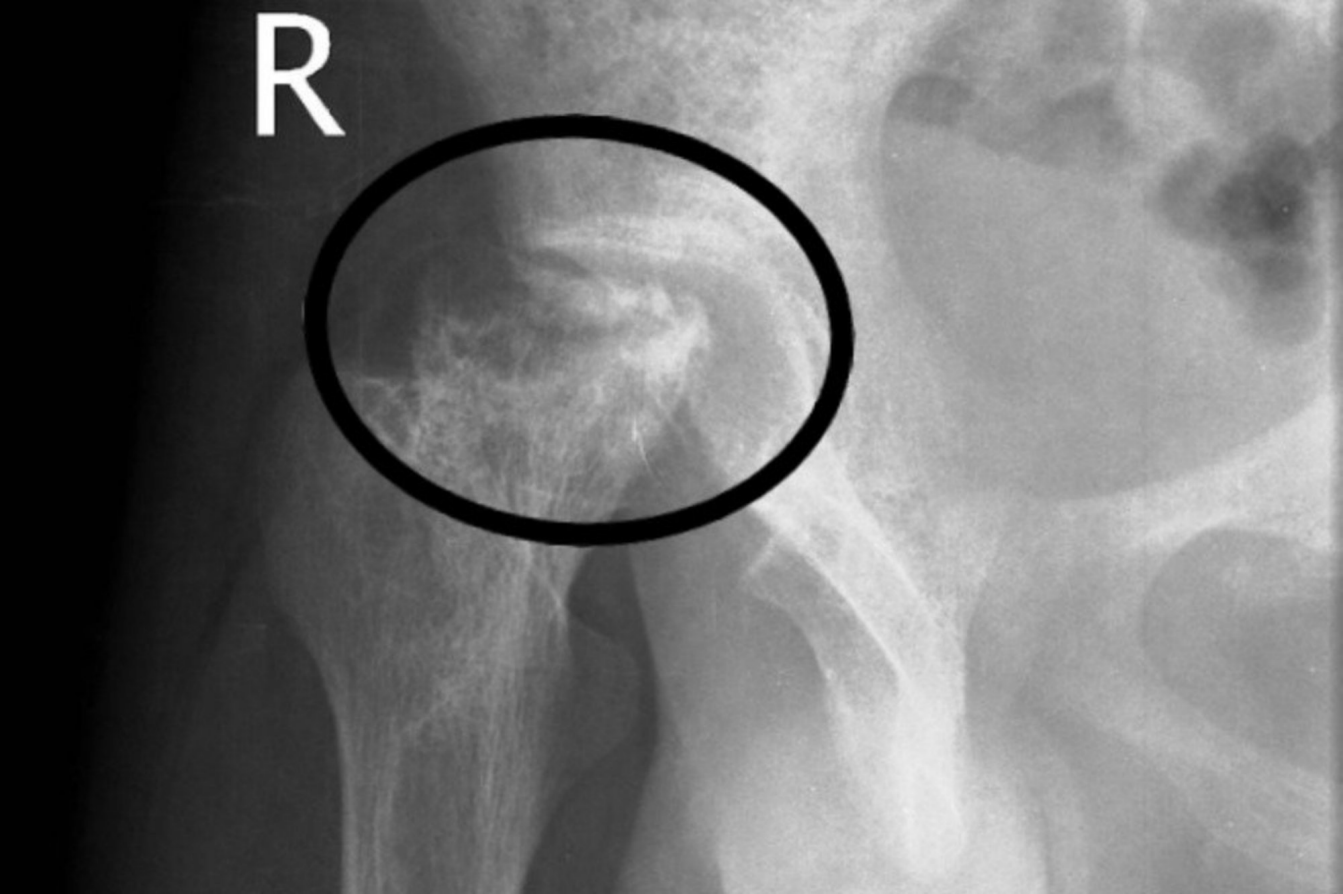
X-ray right hip Advanced osteonecrosis shown in the circle.

On X-rays, subchondral fracture (“crescent sign”) is one of the characteristic features usually present in the later stages of the disease (Figure 2). Advanced stages usually manifest as a degenerative joint disease (Figure 3) [7].

**Figure 4:**
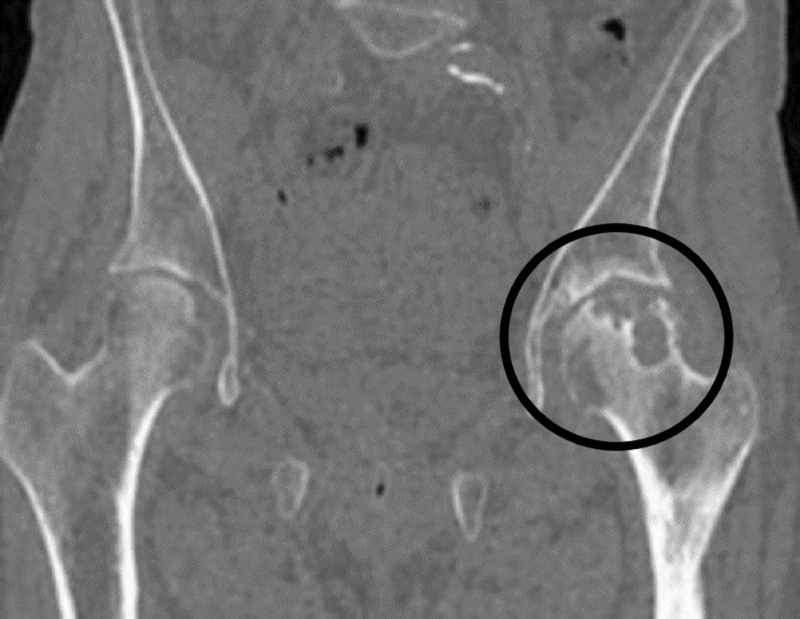
CT pelvis - coronal view Osteonecrosis left hip encircled.

CT is considered the most sensitive test for detecting subchondral fracture of the femoral head. While radiographs and MRIs are useful, a CT delineates the outline of the subchondral bone (Figure 4) [7].

**Figure 5:**
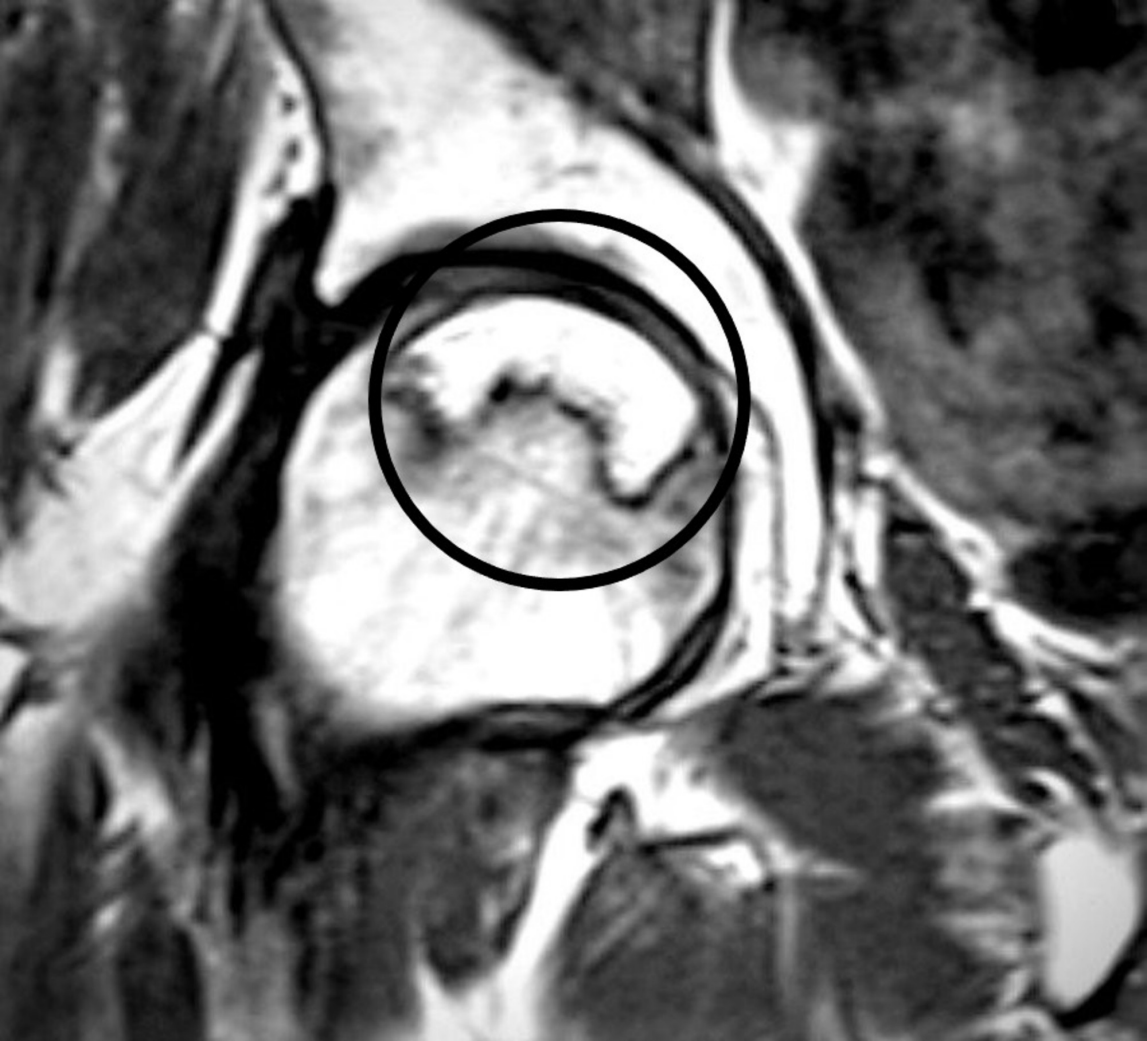
MRI left hip T1 T1 MRI image encircled, showing osteonecrosis in the femoral headband-like lesion. MRI: Magnetic resonance imaging

MRI is the imaging investigation of choice with the highest sensitivity (99%) and specificity (99%) as compared to plain radiographs, CT, or scintigraphy. MRI is the most useful screening tool for early diagnosis and a quantitative evaluation of the disease extent within the femoral head; it is very helpful in the staging of the disease. A single-density “band-like” lesion is seen on T1-weighted images, which is based on a signal from ischemic marrow (Figure 5). There is a “double-line” sign on T2-weighted images representing hypervascular granulation tissue [8].

Classification

In the literature, there are several classification systems used to determine the ON stage for prognosis and assist with treatment decisions. The two most common classifications used in the diagnosis of ONFH are the Ficat and Arlet classification and the Steinberg (University of Pennsylvania) classification [9]. There are three important factors for prognosis: the extent of the osteonecrosis lesion, the location of the lesion within the femoral head, and the presence of bone marrow edema in the proximal femur.

Treatment

Nonsurgical management starts with observation or protected weight bearing. It has a very limited role in the treatment of ONFH except for the follow-up of small asymptomatic lesions until they become symptomatic.

Biophysical modalities have been used as well with limited success, such as extracorporeal shock waves and pulsed electromagnetic fields, but information on their use is sparse.

Enoxaparin may prevent the progression of primary hip ON in patients with thrombophilia or hypofibrinolytic disorders [10]. Alendronate (bisphosphonate) to treat early-stage osteonecrosis was evaluated in two randomized trials, with conflicting results. The theory was the activity of osteoclasts could be inhibited to prevent or delay the collapse of the femoral head [11].

Hyperbaric oxygen therapy is a suggested joint-preserving treatment for symptomatic early-stage ONFH but is especially beneficial in Stage 1 and Stage 2 [12].

The surgical treatment of osteonecrosis can be broadly divided into femoral head-preserving procedures and hip arthroplasty. Femoral head-preserving procedures include core decompression with or without non-vascularised bone grafting, vascularised bone grafting, biologic adjuncts, tantalum rods, and rotational osteotomies.

**Figure 6:**
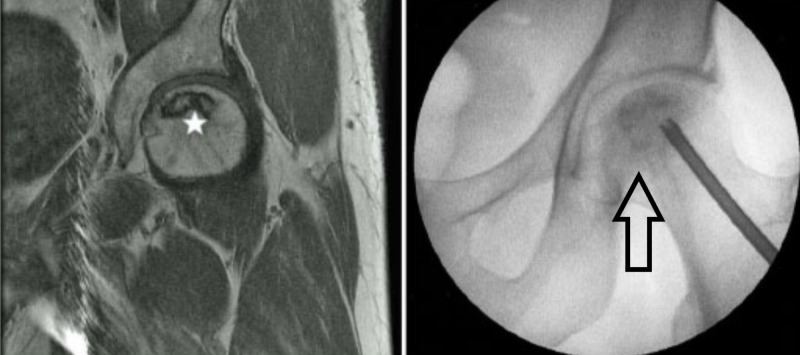
ONFH and decompression MRI with ONFH (left-sided image; star showing the osteonecrosis); X-ray left hip; arrow showing osteonecrosis and decompression. MRI: Magnetic resonance imaging; ONFH: Osteonecrosis of the femoral head

Core decompression has been widely used to treat early-stage ON and is intended to reduce intraosseous pressure in the femoral head, restore vascular flow, and improve pain. The procedure can be performed with a single core tract of varying size or with multiple small core tracts (Figure 6) [13-14].

Core decompression can be supplemented with the insertion of allografts and non-vascularised autografts to provide mechanical support of the osteonecrotic lesion and prevent collapse. Grafting can be performed with the Phemister technique (through the core tract), light bulb technique (through a cortical window at the junction of the cartilage and the femoral neck), or the trap door technique (through a cartilage window) [15].

**Figure 7:**
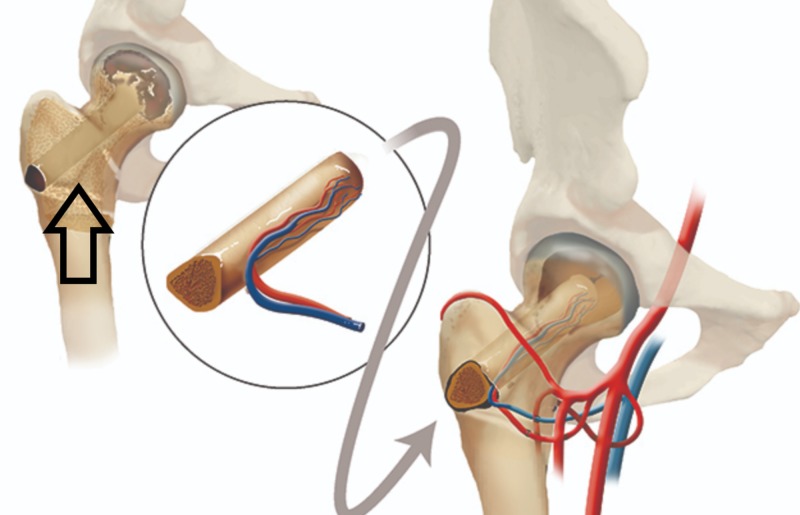
Illustration showing decompression and grafting Post-decompression bone strut using a vascular fibular graft (shown in the circle). (Courtesy: Penn Medicine)

**Figure 8:**
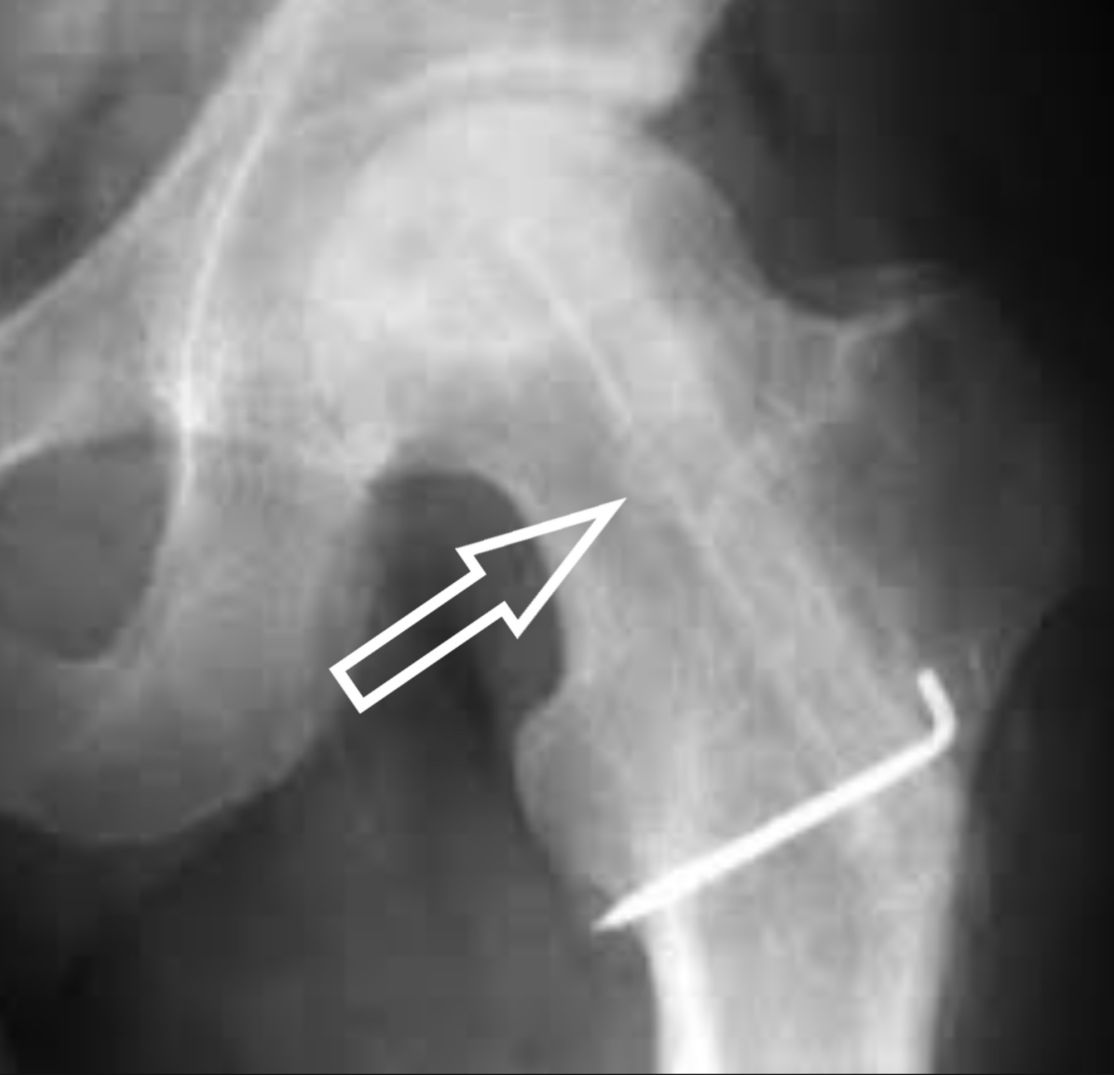
Post-op grafting X-ray X-ray showing bone grafting after decompression with the help of k-wires (Kirschner wires).

For vascularized bone grafting, the fibula or iliac crest graft supports the subchondral bone with a viable, strong bone strut and enhances the revascularization of the femoral head. Free vascularized bone grafting is technically demanding, requires expertise in microsurgery, and is associated with donor site morbidity (Figures 7-8) [16].

**Figure 9:**
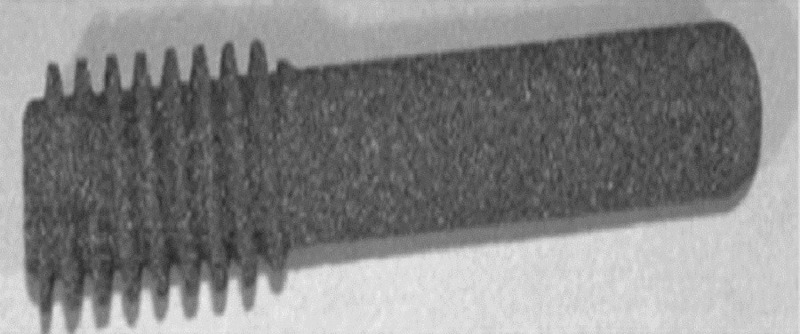
Tantalum rod Tantalum rod in vitro.

**Figure 10:**
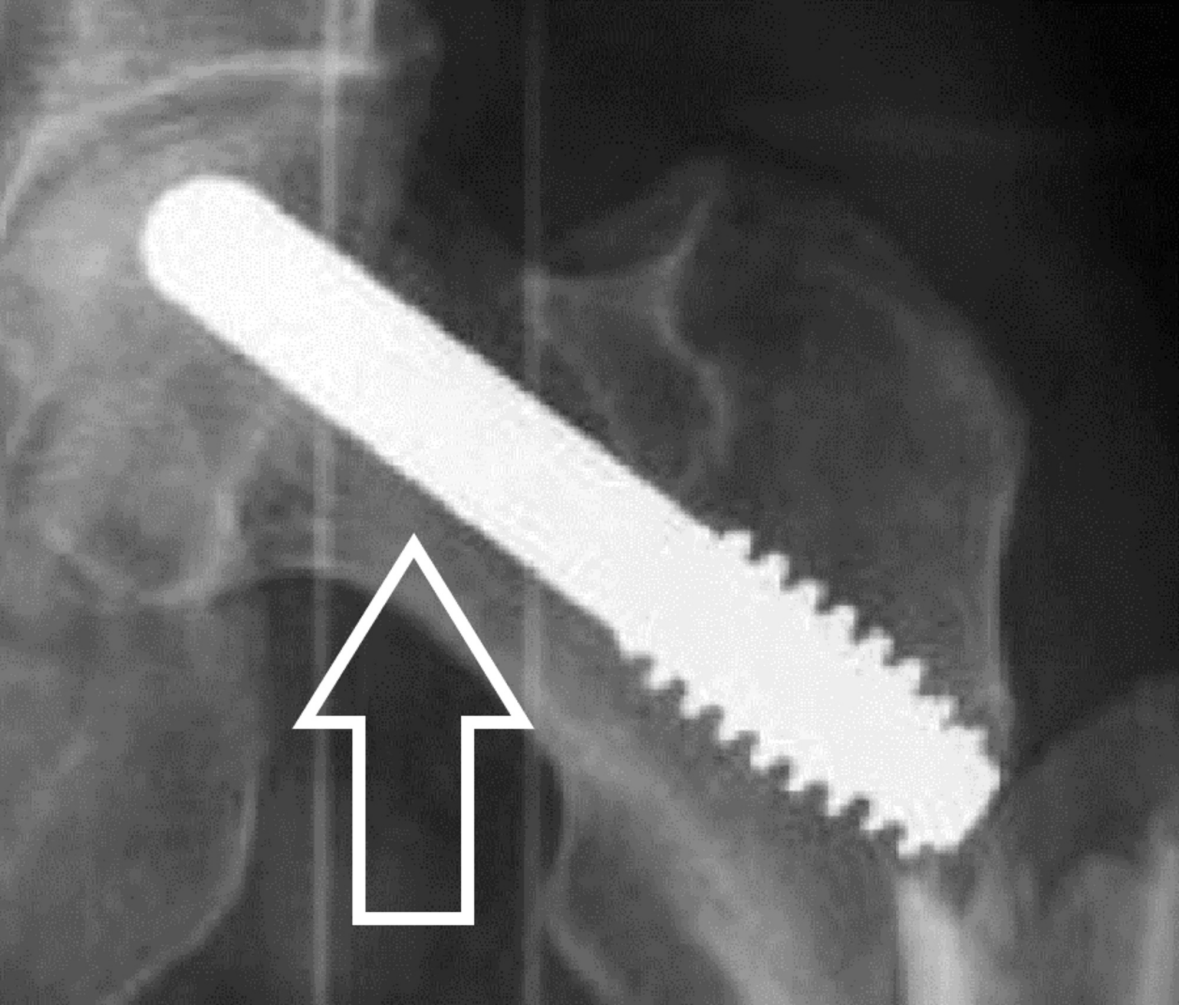
X-ray left hip with Tantalum rod Tantalum rod in vivo highlighted by an arrow.

A tantalum implant has been used as an alternative to bone grafting following core decompression. The tantalum implant should provide mechanical and structural support to the necrotic area, enhanced bone ingrowth given its highly porous nature and osteoconductive micro-texture (Figures 9-10) [17-18].

**Figure 11:**
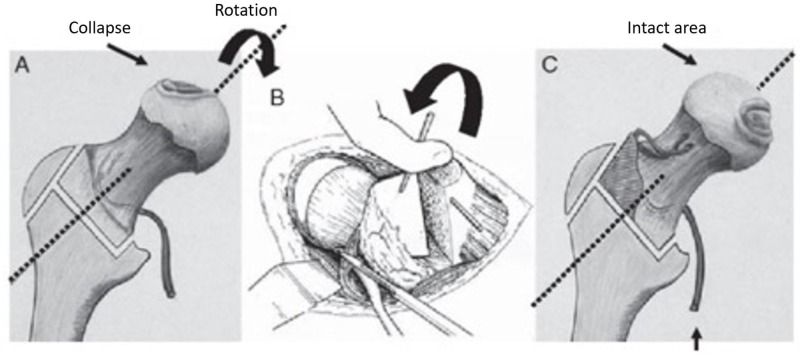
Illustration showing rotation osteotomy The osteotomy is to rotate the damaged weight-bearing surface, so the undamaged area becomes the weight-bearing part of the femoral head. (Courtesy: Nakashima Y, Kubota H, Yamamoto T, Mawatari T, Motomura G, Iwamoto Y: Transtrochanteric rotational osteotomy for late-onset Legg-Calve-Perthes disease. J Pediatr Orthop. 2011, 31:223-228)

Osteotomies are another modality for treatment, with a goal to prevent femoral head collapse by transposing the osteonecrotic area from a weight-bearing to a non-weight-bearing area of the hip joint, thereby diverting mechanical stress from the lesion to healthy bone. Two types of osteotomies have been used: trans-trochanteric rotational osteotomies (anterior or posterior) and intertrochanteric varus or valgus osteotomies. The trans-trochanteric technique is popular in Japan, and its efficacy depends on the size of the osteonecrotic lesion. Trans-trochanteric osteotomies require a sufficiently large area of healthy bone and are technically demanding; the conversion of failed cases to total hip arthroplasty (THA) may be difficult (Figure 11) [19-20].

Total hip replacement is the surgical treatment that most reliably achieves pain relief and provides prompt functional return with a single procedure [21-22]. Patients with ON had a significantly higher dislocation rate than did patients with osteoarthritis because ON patients usually have a better preoperative range of motion compared with a patient with long-standing osteoarthritis. The revision rate in patients younger than age 50 with osteonecrosis was significantly higher compared with that in patients in the same age group with osteoarthritis [23-24].

When preparing an acetabulum in an osteonecrosis hip, the surgeon must remember that the bone quality may be poor secondary to corticosteroid use, lack of weight bearing, or the underlying disease.

Resurfacing arthroplasty preserves bone and does not compromise subsequent conversion to THA. THA may be a viable option in the management of post-collapse ON in young patients with good bone stock [25].

## Conclusions

Patients with symptomatic ON with small lesions should be treated with head-sparing procedures. Patients with large lesions in pre-collapse hips should be treated with head-sparing procedures if the patients are young or have THA in older patients. For patients with a collapsed femoral head, THA is the recommended option.

## References

[1] Choi HR, Steinberg ME, Y Cheng E (2015). Osteonecrosis of the femoral head: diagnosis and classification systems. Curr Rev Musculoskelet Med.

[2] Moya-Angeler J, Gianakos AL, Villa JC, Ni A, Lane JM (2015). Current concepts on osteonecrosis of the femoral head. World J Orthop.

[3] Shah KN, Racine J, Jones LC, Aaron RK (2015). Pathophysiology and risk factors for osteonecrosis. Curr Rev Musculoskelet Med.

[4] Drescher W, Pufe T, Smeets R, Eisenhart-Rothe R v, Jäger M, Tingart M (2011). Avascular necrosis of the hip - diagnosis and treatment [Article in German]. Z Orthop Unfall.

[5] Grose AW, Gardner MJ, Sussmann PS, Helfet DL, Lorich DG (2008). The surgical anatomy of the blood supply to the femoral head: description of the anastomosis between the medial femoral circumflex and inferior gluteal arteries at the hip. J Bone Joint Surg Br.

[6] Gasbarra E, Perrone FL, Baldi J, Bilotta V, Moretti A, Tarantino U (2015). Conservative surgery for the treatment of osteonecrosis of the femoral head: current options. Clin Cases Miner Bone Metab.

[7] Pierce TP, Jauregui JJ, Cherian JJ (2015). Imaging evaluation of patients with osteonecrosis of the femoral head. Curr Rev Musculoskelet Med.

[8] Hu LB, Huang ZG, Wei HY, Wang W, Ren A, Xu YY (2015). Osteonecrosis of the femoral head: using CT, MRI and gross specimen to characterize the location, shape and size of the lesion. Br J Radiol.

[9] Steinberg ME, Steinberg DR (2004). Classification systems for osteonecrosis: an overview. Orthop Clin North Am.

[10] Glueck CJ, Freiberg RA, Sieve L, Wang P (2005). Enoxaparin prevents progression of stages I and II osteonecrosis of the hip. Clin Orthop Relat Res.

[11] Luo R-B, Lin T, Zhong H-M, Yan S-G, Wang J-A (2014). Evidence for using alendronate to treat adult avascular necrosis of the femoral head: a systematic review. Med Sci Monit.

[12] Koren L, Ginesin E, Melamed Y, Norman D, Levin D, Peled E (2015). Hyperbaric oxygen for stage I and II femoral head osteonecrosis. Orthopedics.

[13] Pierce TP, Jauregui JJ, Elmallah RK, Lavernia CJ, Mont MA, Nace J (2015). A current review of core decompression in the treatment of osteonecrosis of the femoral head. Curr Rev Musculoskelet Med.

[14] Steinberg ME, Larcom PG, Strafford B, Hosick WB, Corces A, Bands RE, Hartman KE (2001). Core decompression with bone grafting for osteonecrosis of the femoral head. Clin Orthop Relat Res.

[15] Calori GM, Mazza E, Colombo A, Mazzola S, Colombo M (2017). Core decompression and biotechnologies in the treatment of avascular necrosis of the femoral head. EFORT Open Rev.

[16] Millikan PD, Karas V, Wellman SS (2015). Treatment of osteonecrosis of the femoral head with vascularized bone grafting. Curr Rev Musculoskelet Med.

[17] Varitimidis SE, Dimitroulias AP, Karachalios TS, Dailiana ZH, Malizos KN (2009). Outcome after tantalum rod implantation for treatment of femoral head osteonecrosis. 26 hips followed for an average of 3 years. Acta Orthop.

[18] Liu Y, Yan L, Zhou S, Su X, Cao Y, Wang C, Liu S (2016). Tantalum rod implantation for femoral head osteonecrosis: survivorship analysis and determination of prognostic factors for total hip arthroplasty. Int Orthop.

[19] Kubo Y, Yamamoto T, Motomura G, Karasuyama K, Sonoda K, Iwamoto Y (2016). Patient-reported outcomes of femoral osteotomy and total hip arthroplasty for osteonecrosis of the femoral head: a prospective case series study. SpringerPlus.

[20] Nakashima Y, Kubota H, Yamamoto T, Mawatari T, Motomura G, Iwamoto Y (2011). Transtrochanteric rotational osteotomy for late-onset Legg-Calve-Perthes disease. J Pediatr Orthop.

[21] Baig MN, Baig U, Curtin B (2018). Challenges in hip replacement in hip dysplasia cases and the "happy elephant sign". Cureus.

[22] Baig MN, Dzufar AH, Murphy CG, Curtin B (2018). Intriguing periprosthetic fracture of hip stem and proximal femoral replacement. Cureus.

[23] Kakaria H, Sharma A, Sebastian B (2005). Total hip replacement in avascular necrosis of femoral head. Med J Armed Forces India.

[24] Issa K, Pivec R, Kapadia BH, Banerjee S, Mont MA (2013). Osteonecrosis of the femoral head: the total hip replacement solution. J Bone Joint Surg Br.

[25] Amstutz HC, Le Duff MJ (2016). Hip resurfacing for osteonecrosis: two- to 18-year results of the Conserve Plus design and technique. J Bone Joint Surg Br.

